# Reliability and validity of the Dutch version of the foot and ankle outcome score (FAOS)

**DOI:** 10.1186/1471-2474-14-183

**Published:** 2013-06-11

**Authors:** Inge van den Akker-Scheek, Arnoud Seldentuis, Inge HF Reininga, Martin Stevens

**Affiliations:** 1 Department of Orthopedics, University of Groningen, University Medical Center Groningen, P.O. Box 30.001, , Groningen, 9700 RB, The Netherlands; 2 Department of Traumatology, University of Groningen, University Medical Center Groningen, Groningen, The Netherlands

**Keywords:** Foot, Ankle, Questionnaire, FAOS, Dutch, Reliability, Validity, Orthopaedics

## Abstract

**Background:**

The Foot and Ankle Outcome Score (FAOS) is a patient-reported questionnaire measuring symptoms and functional limitations of the foot and ankle. Aim is to translate and culturally adapt the Dutch version of the FAOS and to investigate internal consistency, validity, repeatability and responsiveness.

**Methods:**

According to the Cross Cultural Adaptation of Self-Report Measures guideline, the FAOS was translated into Dutch. Eighty-nine patients who had undergone an ankle arthroscopy, ankle arthrodesis, ankle ligament reconstruction or hallux valgus correction completed the FAOS, FFI, WOMAC and SF-36 questionnaires and were included in the validity study. Sixty-five of them completed the FAOS a second time to determine repeatability. Responsiveness was analysed in an additional 15 patients who were being treated for foot or ankle problems.

**Results:**

Internal consistency of the FAOS is high (Cronbach’s alphas varying between 0.90 and 0.96). Repeatability can be considered good, with ICC’s ranging from 0.90 to 0.96. Construct validity can be classified as good with moderate-to-high correlations between the FAOS subscales and subscales of the FFI (0.55 to 0.90), WOMAC (0.57 to 0.92) and SF-36 subscales physical functioning, pain, social functioning and role-physical (0.33 to 0.81). Low standard response means were found for responsiveness (0.0 to 0.4).

**Conclusions:**

The results of this study show that the Dutch version of the FAOS is a reliable and valid questionnaire to assess symptoms and functional limitations of the foot and ankle.

## Background

Foot and ankle problems often lead to pain, chronic instability, movement restrictions and ultimately a diminished quality of life [1]. The societal impact of complaints of the musculoskeletal system can be considered high due to the physical limitations that people encounter and the absenteeism from work [2]. This is also reflected in the costs involved for healthcare and society in general. Musculoskeletal disorders account for 6.0% of the cost of healthcare facilities in the Netherlands, where a 14.9% prevalence of foot and ankle problems is reported [2].

Outcome measures are an important means of assessing the patient’s pain and dysfunction associated with a foot or ankle complaint. In recent years more patient-reported questionnaires have been developed that are increasingly used to gain insight into the complaints of patients and the effect of interventions. These patient-reported questionnaires are a valuable addition to the more traditional parameters like physical examination and X-rays, as such parameters do not necessarily need to correlate with the subjective feeling of the patient with respect to e.g. pain, daily functioning and perceived quality of life. Before questionnaires can be used in daily practice or research setting, it is important, however, to study reliability and validity.

The systematic review by Eechaute et al. [3] showed that the Foot and Ankle Outcome Score (FAOS) together with the Functional Ankle Ability Measure (FAAM) and the Foot and Ankle Disability Index (FADI) are good questionnaires to investigate chronic ankle instability. The FAOS was developed in Sweden and is a modification of the Knee Injury and Osteoarthritis Outcome Score (KOOS) [4,5]. The validity and reliability of the original Swedish version of the FAOS as well as of four different translated versions (English, Portuguese [Brazilian], Turkish and Persian [Iran]) is considered good [4,6-8]. However, there is no Dutch version of the FAOS yet. The purpose of this study is (1) to translate the FAOS into the Dutch language and culturally adapt it according to international guidelines, and (2) to investigate internal consistency, validity, repeatability and responsiveness of the Dutch FAOS.

## Methods

### FAOS

The FAOS is an adaptation of the KOOS and aims to measure symptoms and functional limitations of the foot and ankle [4]. The FAOS consists of 42 questions, divided into five different patient-relevant subscales: pain (nine questions); symptoms such as stiffness, swelling and range of motion (seven questions); activities of daily living (17 questions); ability to perform sports and recreational activities (five questions); and foot/ankle-related quality of life (four questions). Answers are given on a five-point Likert scale. Total and subscores are calculated by summing the scores of the individual items. The total score is recoded into a 0–100 scale, with 100 representing no symptoms or limitations.

### Translation

The translation of the questionnaire was done according to the guideline for Cross Cultural Adaptation of Self-Report Measures by Beaton et al. [9]. This guideline is based on the review of Guillemin et al. [10] and are the official guidelines of the American Academy of Orthopaedic Surgeons. The guideline consists of 5 stages: (1) translation, (2) synthesis, (3) back translation, (4) evaluation by a team of experts, and (5) tests. In stage one, the English version of the FAOS was translated into Dutch by two Dutch native speakers who are fluent in English. One person had knowledge of medicine and the FAOS, the other did not. In stage two, both translations were combined by the two translators and a team of experts. In stage three, two persons independently translated the Dutch translation of the FAOS back into English. Both translators were bilingual native English speakers. Neither translator received any background information on the study or the FOAS. With this back-translation the content validity of the questionnaire is warranted. In stage four, the investigator and the same team of experts prepared the final version. In stage five, the Dutch FAOS was tested in eight patients presenting themselves with various foot/ankle problems at the outpatient clinic of the University Medical Center Groningen (UMCG), Groningen, the Netherlands. These patients were asked whether the questions were understandable and if they were able to complete the questionnaire. All patients reported that the questions were understandable and that there were no ambiguities, so no changes to the questionnaire were necessary.

### Participants and procedure

Participants were patients who had undergone an ankle arthroscopy, ankle arthrodesis, ankle ligament reconstruction or hallux valgus correction. All patients were operated at the UMCG. To determine validity, all 185 patients who underwent surgery between January 2007 and December 2010 were sent four questionnaires [FAOS, Foot Function Index (FFI), Western Ontario and McMaster Universities Osteoarthritis Index (WOMAC) and Short Form Health Survey (SF-36)] with an accompanying letter clarifying the purpose and procedure of the study and explaining that return of the questionnaire was taken as consent to participate. The FFI is a reliable patient-reported questionnaire measuring the impact of foot pathology on function and consists of 23 questions on pain, disability and activity restriction [11]. The WOMAC is a valid and reliable self-reported outcome measure for hip and knee osteoarthritis and consists of 24 items on pain, stiffness and function [12,13]. In both the FFI and the WOMAC answers are given on a 5-point Likert scale. Total scores range from 0–100 on the FFI and from 0–96 on the WOMAC. All scores are recoded so that a higher score indicates less pain, disability and activity restriction (FFI) and less pain and stiffness and improved function (WOMAC). The SF-36 is a generic health-related quality of life questionnaire, consisting of 36 items divided into nine subscales [14]. All subscales range from 0–100, with a higher score indicating a better quality of life.

To determine repeatability, respondents were sent the FAOS a second time two to three weeks after completing the first questionnaire. To determine responsiveness of the Dutch FAOS, a separate group of 30 patients who were being treated for foot or ankle problems was sent the FAOS according to the same procedure. Five to six weeks later, participants were asked to complete the FAOS a second time.

Demographic information was retrieved from previous medical documentation of the patient. The study was approved by the Medical Ethical Board of the UMCG. The procedures followed were in accordance with the Helsinki Declaration of 1975, as revised in 2000.

### Data analyses

Descriptive statistics were used to describe main characteristics of the study participants.

#### Internal consistency

Internal consistency is the extent to which items within a subscale are homogeneous, thus measuring the same construct [15,16]. Cronbach’s alpha was used to evaluate internal consistency of the FAOS. A Cronbach’s alpha between 0.70 and 0.95 indicate an acceptable level of internal consistency [16].

#### Validity

As no gold standard exists, construct validity of the FAOS was assessed by determining the relationship between the FAOS and the FFI. Additionally, the relationship between the FAOS and the SF-36 and between the FAOS and the WOMAC was determined, in line with previous studies into the FAOS [6,7]. Data were tested for normality using the Shapiro-Wilk test and normal Q-Q plot. As some variables were not normally distributed, Spearman’s correlation coefficients were calculated to assess construct validity. Correlation coefficients above 0.6, 0.6 to 0.3 and less than 0.3 are considered high, moderate and low, respectively [17].

#### Repeatability

For repeatability, both absolute agreement and test-retest reliability were examined. Degree of absolute agreement was determined with a Bland & Altman analysis [18]. Mean difference between test and re-test score with corresponding 95% confidence interval (CI) was calculated. When zero lies within the 95% CI this can be seen as a criterion for absolute agreement. If zero falls outside the 95% CI, this is an indication for a bias in the measurements. Further, the standard error of measurement (SEM) was calculated as a measure of absolute measurement error of the questionnaire [15]. To calculate the SEM, the standard deviation of the mean difference between both measurements was divided by √2 [19].

Evaluation of the test-retest reliability was performed using the intraclass correlation coefficient (ICC) with corresponding 95% CI. An ICC two-way random effects model, type agreement, was used. An ICC above 0.7 is considered good [16,20].

#### Responsiveness

Responsiveness is the extent to which a questionnaire is able to detect changes over time. To gain insight into responsiveness, the Standardized Response Means (SRM) with corresponding 95% CIs were calculated for each subscale of the FAOS. These effect estimates were interpreted according to Cohen: a SRM of 0.2 to 0.4 is considered a small effect, 0.5 to 0.7 as moderate and higher than 0.8 as large [21]. All statistical analyses were performed with the Statistical Package for Social Sciences (SPSS, version 18).

## Results

Of the 185 patients who were sent an invitation to participate, 103 patients responded (response rate 56%). Of these respondents, 12 indicated being unwilling to participate in the study and in two cases it were family members who responded informing us that the patients were deceased. This left 89 patients who could be contacted a second time in order to determine repeatability; 65 of them completed the FAOS a second time (response rate 73%). To determine responsiveness 30 patients were invited; 22 patients responded (response rate 73%) the first time, and 15 of them completed the FAOS twice (response rate 68%). Demographic characteristics are displayed in Table 1.

**Table 1:** Demographic characteristics

	**Validity**	**Repeatability**	**Responsiveness**
	**N = 89**	**N = 65**	**N = 15**
Age in years [mean ± SD (range)]	49 ± 17 (17–80)	51 ± 17 (19–80)	40 ± 19 (16–69)
Male [N (%)]	25 (28)	17 (26)	4 (27)
Treatment [N (%)]			
Ankle arthrodesis	14 (15.7)	10 (15.4)	2 (13.3)
Ankle arthroscopy	13 (14.6)	11 (16.9)	1 (6.7)
Ankle ligament reconstruction	8 (9.0)	5 (7.7)	1 (6.7)
Hallux Valgus correction	54 (60.7)	39 (60.0)	11 (73.3)

### Internal consistency

Cronbach’s alpha was 0.90 for the pain subscale, 0.93 for the symptoms and ADL subscales, 0.94 for the sports and recreational activities subscale, and 0.96 for the quality of life subscale.

### Construct validity

The correlations between the FAOS subscales and the subscales of the FFI, WOMAC and SF-36 are presented in Table 2.

**Table 2:** Construct validity of the Dutch FAOS – Spearman’s correlation coefficients between FAOS and FFI, WOMAC and SF-36

	**FAOS**				
	**Symptoms**	**Pain**	**ADL**	**Sports & recreational activity**	**Quality of life**
**FFI**					
Pain	0.56	0.90	0.86	0.71	0.78
Disability	0.62	0.82	00.89	0.84	0.84
Activity restriction	0.55	0.71	0.80	0.76	0.74
**WOMAC**					
Pain	0.57	0.89	0.90	0.74	0.78
Stiffness	0.65	0.72	0.73	0.66	0.74
Function	0.58	0.79	0.92	0.82	0.75
**SF-36**					
Physical functioning	0.53	0.62	0.79	0.71	0.62
Social functioning	0.33	0.52	0.63	0.42	0.48
Role-physical	0.37	0.51	0.60	0.53	0.48
Role-emotional	0.11	0.21	0.36	0.24	0.21
Mental health	0.12	0.15	0.20	0.26	0.28
Vitality	0.27	0.46	0.54	0.51	0.50
Pain	0.58	0.75	0.81	0.77	0.72
General health	0.19	0.45	0.52	0.34	0.33
Health change	0.14	0.42	0.44	0.42	0.38

#### Comparison between FAOS and FFI

Spearman correlations were moderate to high (0.55 to 0.90) between the subscales of the FAOS and the FFI. Moderate-to-high correlations were seen with the FAOS subscale symptoms. All other correlations were high (see Table 2).

#### Comparison between FAOS and WOMAC

Spearman correlations were moderate to high (0.57 to 0.92) between the subscales of the FAOS and the WOMAC. Moderate-to-high correlations were seen with the FAOS subscale symptoms. All other correlations were high (see Table 2).

#### Comparison between FAOS and SF-36

Spearman correlations were low to high (0.11 to 0.81) between the subscales of the FAOS and the SF-36. Moderate-to-high correlations were found between the FAOS subscales and the SF-36 subscales pain, physical functioning, social functioning and role-physical. Low-to-moderate correlations were found between the FAOS subscales and the SF-36 subscales mental health and role-emotional (see Table 2).

### Reliability

Table 3 shows the mean difference between the first and second measurement, and the intraclass correlation coefficient (ICC) for each subscale of the FAOS. ICCs were high for all subscales ranging from 0.90 to 0.96. The 95% CI of the mean difference in the subscale quality of life does not contain zero, indicating a systematic bias between the first and second meeting. The standard error of measurement (SEM) of the subscales ranged between 7.6 and 10.6.

**Table 3:** Reliability of the Dutch FAOS

	**1st assessment mean (SD)**	**2nd assessment mean (SD)**	**Mean difference (95% CI)**	**SEM**	**ICC (95% CI)**
Symptoms	67.7 (24.0)	68.5 (24.3)	−0.8 (−3.9,2.5)	9.1	0.93 (0.88, 0.96)
Pain	69.8 (22.9)	70.3 (25.0)	−0.5 (−3.6,3.6)	10.0	0.90 (0.84, 0.94)
ADL	77.2 (22.6)	76.1 (25.2)	1.1 (−1.7,4.6)	8.9	0.93 (0.88, 0.96)
Sports & recreational activity	55.9 (34.0)	54.2 (32.4)	1.7 (−5.4,2.5)	10.6	0.95 (0.91, 0.97)
Quality of life	51.1 (28.9)	53.0 (28.2)	−1.9 (−5.7,-0.3)	7.6	0.96 (0.94, 0.98)

### Responsiveness

Table 4 shows the responsiveness of the Dutch FAOS. All subscales have low SRMs ranging from 0.01 to 0.36.

**Table 4:** Responsiveness of the Dutch FAOS

	**1st measurement**	**2nd measurement**	**Mean difference**	**SRM**
	**mean (SD)**	**mean (SD)**	**(SD)**	
Symptoms	61.0 (22.6)	61.2 (25.1)	0.2 (20.9)	0.0
Pain	58.3 (22.4)	61.1 (22.8)	2.8 (16.5)	0.2
ADL	67.6 (24.2)	70.4 (22.9)	2.8 (13.8)	0.2
Sports & recreational activity	35.2 (23.9)	43.0 (27.1)	7.7 (21.2)	0.4
Quality of life	38.9 (25.7)	40.4 (23.8)	1.5 (16.3)	0.1

## Discussion

The results of this study show that the Dutch version of the FAOS is a reliable and valid questionnaire to assess symptoms and functional limitations of the foot and ankle.

The internal consistency of the Dutch FAOS can be considered high (Cronbach’s alphas varying between 0.90 and 0.96). This means that the consistency of questions within the subscales is good, indicating that homogeneous constructs are being measured. These results are similar to the original (0.88 to 0.97) [4], the Turkish (0.79 to 0.97) [7] and the Portuguese (0.82 to 0.96) [8] versions of the FAOS. In all versions, including the Dutch version, the highest Cronbach’s alphas were found for the Activities of Daily Living (ADL) subscale.

The construct validity of the Dutch FAOS can be classified as good. Construct validity was determined by comparing the FAOS with two other disease-specific questionnaires (FFI and WOMAC) and a generic questionnaire (SF-36). Moderate-to-high correlations were found between the Dutch FAOS and the respective subscales of the FFI and WOMAC. These results indicate that the FAOS is an appropriate outcome measure to assess the functional status of patients with foot/ankle problems. A comparison of these correlations with previous versions of the FAOS is not possible, as construct validity of other language versions of the FAOS was not determined by means of the FFI or WOMAC. In the original Swedish version of the FAOS an indication of construct validity was obtained by comparing the FAOS with the Karlsson score [22]. The Karlsson score is a disease-specific questionnaire completed by the patient to evaluate function of the ankle, and includes the subscales pain and other symptoms, activities of daily living, functional abilities for sports and recreational activities, and quality of life. Results showed moderate-to-high (0.58-0.67) correlations between the subscales of the FAOS and those of the Karlsson score. These results are in the same order of magnitude as the ones we found for construct validity, even though the range is smaller.

When comparing the FAOS with the generic SF-36 questionnaire, moderate-to-high correlations were found between the FAOS and the SF-36 subscales physical functioning, pain, social functioning and role-physical. Correlation between the FAOS and the SF-36 subscales mental health and role-emotional showed low-to-moderate coefficients. These differences in correlations between the FAOS and the various subscales of the SF-36 can be explained by the fact that a disease-specific and a generic questionnaire were compared. Subscales from both lists that measure similar constructs lead to a high correlation (convergent validity), and subscales that measure different constructs lead to lower correlations (divergent validity). The Turkish and Persian versions of the FAOS were also compared with related subscales of the SF-36. This comparison led to corresponding results in the Turkish and Dutch versions (0.42 to 0.78) [7], and to lower correlations in the Persian version (−0.33 to 0.58) [6]. Construct validity was not determined in other versions of the FAOS.

The repeatability of the Dutch FAOS can be considered good, with ICC’s varying between 0.90 and 0.96. These results are in line with those of the original [4], the Persian [6], the Turkish [7] and the Portuguese [8] versions of the FAOS. The degree of absolute agreement can be considered good for the subscales symptoms, pain, ADL function and sports and recreational activity. Within the subscale foot/ankle-related quality of life a small bias is seen which seems to be caused by two questionnaires with a large deviation from the mean. After removing both questionnaires and recalculating the 95% CI for the mean, zero lies within the 95% CI. A reason for these outliers could not be found. As repeatability of the FAOS in other languages has not been assessed, the findings for the Dutch FAOS could not be compared. The SEM varies between 7.6 and 10.6. Whether this should be interpreted as a small or a large measurement error depends on the minimally important change, which is not yet determined for the FAOS.

With respect to responsiveness it can be concluded that low standard response means were found (0.0 to 0.4). A low SRM means that the questionnaire is not able to detect changes in people’s health adequately [21]. A possible explanation for the low SRMs in this study can be the fact that our participants had already undergone foot/ankle surgery when filling in the FAOS for the first time. It is logical to expect the largest improvement to be seen in the period before and after surgery; this is consequently reflected in larger SRMs. The average difference in all subscales of the Dutch FAOS being low with high standard deviations supports this explanation. In conclusion, these results show that the FAOS in this study is not able to detect small changes. A comparison with the other-language versions of the FAOS is not possible, as responsiveness was not analysed.

It is important to note that our research was not without limitations. Firstly, not all patients were willing to participate. The response rate after the first mailing was 56%. Secondly, the diversity in foot/ankle problems is large and thus not all problems were represented in our research population. However, in comparison with the original version of the FAOS, in which only lateral ankle ligament reconstructions were enrolled, this study included a greater diversity of foot/ankle problems.

## Conclusions

The results of this study show that the FAOS was culturally adapted and translated into a Dutch-language version successfully. Internal consistency, repeatability and construct validity can all be considered good, implying that the Dutch version of the FAOS is a reliable and valid questionnaire to assess symptoms and functional limitations of the foot and ankle. For future research it is recommended to further determine responsiveness in a patient population with a first assessment before surgery.

## Competing interests

The authors declare that they have no competing interests.

## Authors’ contributions

IvdAS participated in the design and coordination of the study, performed the data analysis, and drafted the manuscript. AS carried out the data collection, performed the data analysis and drafted the manuscript. IHFR participated in the data analysis and critically revised the manuscript. MS participated in the design and coordination of the study and drafted the manuscript. All authors read and approved the final version of the manuscript.

## Authors’ information

IvdAS, PhD, is a human movement scientist and a senior researcher at the department of Orthopedics of the University Medical Center Groningen, the Netherlands. AS, MD, is a basic medical doctor with interest in orthopedics. IHFR, PhD, is a human movement scientist and a research coordinator at the department of Traumatology of the University Medical Center Groningen, the Netherlands. MS, PhD, is a human movement scientist and a research coordinator at the department of Orthopedics of the University Medical Center Groningen, the Netherlands.

## Pre-publication history

The pre-publication history for this paper can be accessed here:

http://www.biomedcentral.com/1471-2474/14/183/prepub
